# SVD-Based Evaluation of Multiplexing in Multipinhole SPECT Systems

**DOI:** 10.1155/2008/769195

**Published:** 2008-12-21

**Authors:** Aaron K. Jorgensen, Gengsheng L. Zeng

**Affiliations:** ^1^Department of Electrical and Computer Engineering, University of Utah, Salt Lake City, UT 84112, USA; ^2^Department of Radiology, University of Utah, Salt Lake City, UT 84108, USA

## Abstract

Multipinhole SPECT system design is largely a trial-and-error process. General principles can give system designers a general idea of how a system with certain characteristics will perform. However, the specific performance of any particular system is unknown before the system is tested. The development of an objective evaluation method that is not based on experimentation would facilitate the optimization of multipinhole systems. We derive a figure of merit for prediction of SPECT system performance based on the entire singular value spectrum of the system. This figure of merit contains significantly more information than the condition number of the system, and is therefore more revealing of system performance. This figure is then compared with simulated results of several SPECT systems and is shown to correlate well to the results of the simulations. The proposed figure of merit is useful for predicting system performance, but additional steps could be taken to improve its accuracy and applicability. The limits of the proposed method are discussed, and possible improvements to it are proposed.

## 1. INTRODUCTION

Small-animal SPECT imaging provides the opportunity for advanced monitoring and analysis of cancer drug tests in laboratory animals. In order to be effective, a small-animal SPECT system must have high spatial resolution and high sensitivity. The design of multipinhole systems involves many subtle factors which affect both resolution and sensitivity in ways that are difficult to model. Currently, systems are designed based only on general principles; optimization is not a part of the design procedure. Once an aperture is designed, it is tested and analyzed. A method whereby system performance could be predicted, and therefore optimized, in the design phase would allow system designers to experiment with a wider range of design possibilities and to achieve better design results overall.

The main problem in deriving a system performance predictor is the definition of system performance. An optimal system obtains a balance between high spatial resolution and low system noise. Therefore, an objective error predictor must favor both system characteristics equally; an optimal system, as defined by the error predictor, must give low noise and allow for detection of small lesions. We present an error predictor which is shown to account for both spatial resolution and noise, and therefore correlates to image quality in terms of usefulness to the clinician.

In addition, the error predictor provides an objective measure of system performance. Current evaluations of SPECT systems include simulation and actual physical imaging. Of those performing physical experiments, some use laboratory animals [1, 2], some use phantoms [3–5], and some use both [6, 7]. Some of those using simulation to evaluate their systems image a single point or a homogeneous sphere, instead of the type of complex system that would be encountered in clinical use. In addition, current evaluation methods are not completely thorough or standardized, either. Systems can be evaluated in terms of signal-to-noise ratio (SNR) [1], contrast-to-noise ratio, mean square error (MSE) [4, 5], or other performance indicators [3, 8]. Others give no quantitative results and rely on visual comparison of results [2, 7, 9].

In order to obtain this error prediction, the singular value spectrum must be calculated. The matrix-based representation of clinical systems is far too large to store the entire system matrix in the computer memory. We show that the application of the power method to the analysis of SPECT imaging systems is valuable because of the ability to use simulation to find the singular value spectrum of a system. This allows for a frequency-based analysis of systems involving attenuation, photon scattering, and other complex and random phenomena, for which the creation of a system matrix would be complicated.

The theoretical background of the proposed error estimate is presented in Section 2. The generalized SPECT system is presented as a matrix algebra problem. The singular value decomposition is used to analyze the system in terms of frequency content. The relationship between iteration of the minimal-residual (MR) algorithm and frequency content of the reconstructed image is discussed briefly. The singular-value-based analysis is used to create an estimate of the error inherent in the imaging system. The power method is explained and used to determine singular values of the system.

In Section 3, experimentation is presented to verify the error estimate derived in Section 2. Methodology is presented for a two-dimensional and a three-dimensional SPECT simulation. A two-dimensional and a three-dimensional phantom are imaged to a noisy detector and the projections are reconstructed. The results of these simulations are analyzed in terms of error, and these error measurements are compared to the error estimates. The results are discussed, and the efficacy of the error estimate is examined.

Section 4 presents a summary of the work and the conclusions drawn from the results. Possibilities for future work are discussed, including areas into which research must be extended to qualify the proposed error estimate for use in design of clinical systems.

## 2. THEORY

### 2.1. Matrix representation of projection-backprojection

For any given system, a matrix **B** can be defined, which defines the translation of the object **x** (arranged as a column vector of length *m*) to a set of projections **p**:

(1) Bx = p.

Several algorithms exist for solving this type of problems [10–12]. However, for an ill-posed problem, **Bx** = **p** is usually inconsistent due to noise and a solution does not exist. To solve this problem, let us define the transposed matrix **B** ^*T*^ as the backprojection operation, that is, a map from the projection set **p** to the backprojected image vector **B** ^*T*^ **p**. A projection-backprojection matrix can then be defined as

(2) $$A\;=\;B^{T}B,$$

and image reconstruction can be performed by solving

(3) $$Ax\;=\;B^{T}p.$$

In this form, the problem is always consistent, and a unique, least-squares solution can be found, which is also the least-squares solution of **Bx** = **p**. An evaluation of the imaging system can be performed by examining pertinent characteristics of **A**. Such a figure of merit would provide an objective means by which multipinhole apertures could be evaluated and optimized.

### 2.2. Singular value decomposition of system matrix

The singular value decomposition (SVD) of the projection-backprojection matrix **A** can be represented as

(4) $$A\;=\;U\Lambda U^{T}.$$

Because **U** is a unitary matrix, **U** ^*T*^ **U** = **I**, or **U** ^*T*^ = **U** ^−1^. Thus, if all singular values are nonzero, we have

(5) $$A^{-1}\;=\;{\left[U\Lambda U^{T}\right]}^{-1}\;=\;{\left[U^{T}\right]}^{-1}\Lambda^{-1}U^{-1}\;=\;U\Lambda^{-1}U^{T}.$$

This can be used to solve the original problem:

(6) $$\begin{array}{c} Ax\;=\;B^{T}p,\\ x\;=\;A^{-1}B^{T}p\;=\;U\Lambda^{-1}U^{T}B^{T}p. \end{array}$$

From the singular value decomposition, **Λ** is a diagonal matrix which contains, in descending order, the singular values of **A**, which are all nonnegative:

(7) $$\begin{array}{ll} \Lambda & =[\begin{array}{ccccc} \lambda_{1} & 0 & \cdots & \cdots & 0\\ 0 & \lambda_{2} & 0 & \cdots & 0\\ \vdots & 0 & \ddots & \ddots & \vdots \\ \vdots & \vdots & \ddots & \ddots & 0\\ 0 & 0 & \cdots & 0 & \lambda_{m} \end{array}],\\ \Lambda^{-1} & =[\begin{array}{ccccc} \frac{1}{\lambda_{1}} & 0 & \cdots & \cdots & 0\\ 0 & \frac{1}{\lambda_{2}} & 0 & \cdots & 0\\ \vdots & 0 & \ddots & \ddots & \vdots \\ \vdots & \vdots & \ddots & \ddots & 0\\ 0 & 0 & \cdots & 0 & \frac{1}{\lambda_{m}} \end{array}]. \end{array}$$

For any imaging system, there is therefore a threshold for acceptable values of *λ*_*k*_. Values of *λ*_*k*_ below this threshold will add more noise to the system than image data, and thus all values of *λ*_*k*_ below the threshold are truncated, as in (8). The threshold is defined by the variable *n*, such that all inverted singular values corresponding to *k* > *n* are set to zero. We define

(8) $${\overset{˜}{\Lambda }}^{-1}\;=\;\left[\begin{array}{cccccc} \frac{1}{\lambda_{1}} & 0 & & \cdots & & 0\\ 0 & \ddots & & & & \vdots \\ & & \frac{1}{\lambda_{n}} & \ddots & & \vdots \\ \vdots & & \ddots & 0 & & \vdots \\ & & & & \ddots & 0\\ 0 & & \cdots & & 0 & 0 \end{array}\right].$$

It follows that for **U** = [**U**_1_, **U**_2_,…, **U**_*m*_] and **U**_*k*_ = [*u*_1,*k*_, *u*_2,*k*_,…, *u*_*m*,*k*_]^*T*^ we can define a generalized inverse of **A** as

(9) $$\begin{array}{ll} A^{†} & =U\overset{˜}{\Lambda }U^{T}\\ & =\left[\begin{array}{ccccccc} \frac{U_{1}}{\lambda_{1}} & \frac{U_{2}}{\lambda_{2}} & \cdots & \frac{U_{n}}{\lambda_{n}} & 0 & \cdots & 0 \end{array}\right]\left[\begin{array}{c} U_{1}^{T}\\ U_{2}^{T}\\ \vdots \\ U_{m}^{T} \end{array}\right]\\ & =\frac{1}{\lambda_{1}}U_{1}U_{1}^{T}+\frac{1}{\lambda_{2}}U_{2}U_{2}^{T}+\cdots +\frac{1}{\lambda_{n}}U_{n}U_{n}^{T}. \end{array}$$

The regularized solution **x**′ can thus be written as the following summation:

(10) $$x^{\prime }\;=\;\overset{n}{\underset{k=1}{\sum }}\;\frac{1}{\lambda_{k}}U_{k}U_{k}^{T}B^{T}p.$$

### 2.3. Noise analysis

The reconstructed image **x**′ is expressed as a linear combination of image components **U**_*k*_ **U**_*k*_^*T*^ **B** ^*T*^ **p**, with scaling factors 1/*λ*_*k*_. As in a Fourier transform, each of these image components contains frequency content of the total image **x**′, with frequency increasing with *k*.

**Figure 1:**
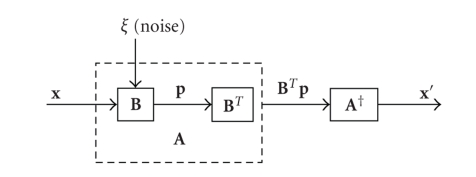
Block diagram of projection-backprojection operation. Noise is introduced at the projection operation, **B**. Image components which are “attenuated” in **B** and **B** ^*T*^ must be “amplified” in **A** ^†^, increasing the noise in **x**′.

The projection-backprojection and reconstruction process can be visualized as in Figure 1. In this conceptual system, a noise signal ***ξ*** is introduced in the projection operation. The image components are “filtered” in the **A** operation by the corresponding scaling factors *λ*_*k*_. Each component must therefore be amplified by a factor of 1/*λ*_*k*_ in the inverse operation, **A** ^†^. The noise in **x**′ is therefore a function of the noise ***ξ*** and the singular values *λ*_*k*_ in **A** ^†^.

In SPECT, the projection data noise is Poisson distributed, that is, its variance equals its mean. We can assume that in the backprojected image, the noise ***ξ*** is Poisson distributed. Let *N* be the total photon count in a projection data set, then the signal uncertainty, or the square root of the noise variance divided by the mean, can be approximated as

(11) $$\frac{\sqrt{N}}{N}\;=\;\frac{1}{\sqrt{N}}.$$

Considering that the noise power of each image component in (10) is

(12) $$\frac{1}{\lambda_{k}^{2}},$$

if we assume that the noise power is uniformly distributed over the entire singular-value-decomposition spectrum, then the susceptibility of the system to noise is the Poisson noise uncertainty multiplied by the sum of the noise powers

(13) $$\frac{1}{\sqrt{N}}\sqrt{\overset{n}{\underset{k=1}{\sum }}\;\frac{1}{\lambda_{k}^{2}}},$$

which is hereafter referred to as the “noise amplification factor.” Because it involves all singular values, this error estimate describes the general ability of a system to reproduce image data at all frequencies.

A less accurate but less computationally expensive estimate of the noise amplification involves the condition number *K*(**A**), the ratio of the largest to the smallest singular value of **A**. In this way, the condition number can be calculated for a real-world system and the uncertainty can be estimated as

(14) $$\frac{K(A)}{\sqrt{N}}.$$

Although not as precise as the noise amplification factor, the condition number does relate to how well-posed the problem **Ax** = **B** ^*T*^ **p** is. However, systems with different singular value spectra can have identical condition numbers, even though their performance is not the same. For this reason, the noise amplification factor is a more revealing estimate of system performance.

### 2.4. Power method

For real-world systems, it is not feasible to obtain singular values from **A** due to its large size. The power method is an iterative algorithm which can estimate the dominant singular value of a system indirectly; only access to the matrix operation is needed [13]. The *method of deflation* is used to find nondominant singular values, that is, the second singular value of **A** is equal to the dominant singular value of

(15) $$A_{2}\;=\;A\;-\;\lambda_{1}U_{1}U_{1}^{T},$$

and so on, so that the *k*th singular value of **A** is found by estimating the dominant singular value of

(16) $$A_{k}\;=\;A_{k-1}\;-\;\lambda_{k-1}U_{k-1}U_{k-1}^{T},$$

Of course, for large systems, access to **A** is not available, so an equivalent operation—simulation of the projection and backprojection operations—is performed on the vectors employed in the power method algorithm in order to compute singular values. For an image of size *N* × *N* × *N*, the computational complexity of the projection (or backprojection) is *O*(*N* ^3^) per projection, or *O*(*N* ^4^) for the entire image, assuming that the number of projections is *O*(*N*). Therefore, the computational complexity of computing a condition number (two terms) with the power method is *O*(*N* ^4^), and the complexity of computing the entire noise amplification factor (*N* ^3^ terms) with the power method is *O*(*N* ^7^). It is not realistic to compute an entire SVD spectrum for each system to be analyzed. In practice, only a small number of singular values are computed, so that the overall computational complexity remains *O*(*N* ^4^). On our 2 GHz Windows-based computer, a MATLAB-based projection-backprojection operation requires approximately two minutes to run. In order to be useful for calculations of many SVD spectra, the simulation would have to be optimized, but this was not done for this paper.

## 3. EXPERIMENTS AND RESULTS

### 3.1. Setup

Preliminary simulations were run for a two-dimensional phantom with a one-dimensional detector. The phantom was a 31 × 31 pixel modified Shepp-Logan phantom. The phantom was imaged at 120 angles to a 60-pixel detector, using apertures of 1, 3, 5, 7, 9, 11, 13, and 15 evenly spaced pinholes. Poisson noise was added to the projections, and the images were reconstructed using the MR algorithm.

The MR algorithm is used in place of the more popular ML-EM algorithm because of its natural applicability to the singular value decomposition. Although the ML-EM algorithm models Poisson noise properly [14, 15], it cannot be analyzed with a simple algebraic method, and so is not suitable for this analysis. Under the assumption that photon count per detector bin is sufficiently high (greater than 10), the Poisson noise can be approximated as Gaussian, and so the MR algorithm can be used.

Because of the relatively small system size, the errors in these images were compared to two error predictors: one based on the condition number of the system, and the other based on the *noise amplification factor*, which is based on the entire singular-value spectrum. As discussed earlier, a function of the condition number *K*(**A**) can be substituted for the noise amplification factor. The square of the condition number, (*K*(**A**))^2^, seems to be a good estimate of the noise amplification factor, and so the condition-number-based error estimate used in these experiments is ${\left(K(A)\right)}^{2}/\sqrt{N}$. This is, of course, an empirical fit and not based on any rigorous mathematical principle.

**Figure 2:**
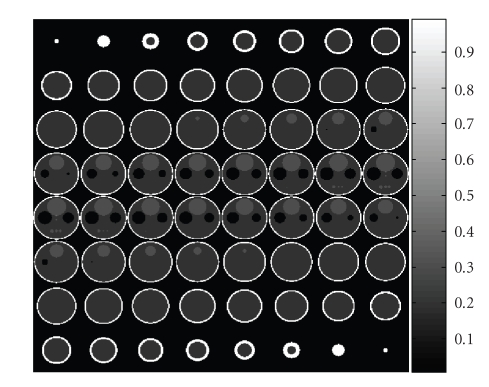
Custom phantom used in simulation.

**Figure 3:**
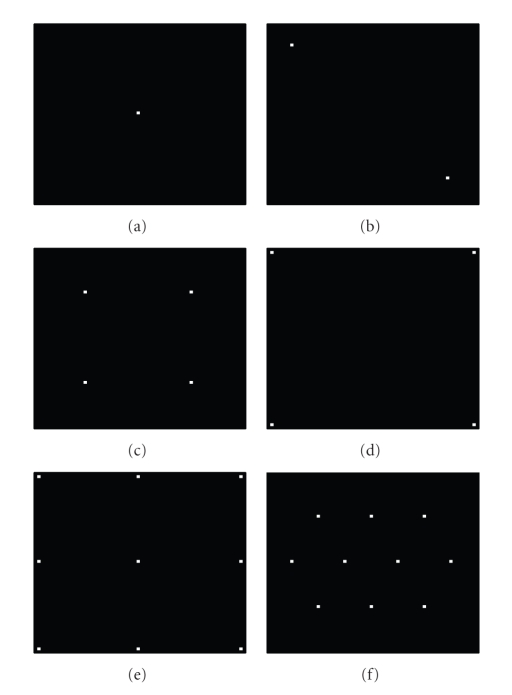
Apertures used in final simulation.

Final simulations were run for a three-dimensional phantom with a two-dimensional detector. The phantom used (Figure 2) is a custom 64 × 64 × 64 voxel phantom, which has been made to resemble the Shepp-Logan phantom. The Shepp-Logan phantom is used frequently in analysis of medical imaging systems, and is designed to resemble a head. The phantom was imaged at 60 angles to a 128-by-128 pixel detector through apertures with varying numbers of holes and varying arrangements of holes. The apertures are illustrated in Figures 3(a)–3(f), and will be referred to as apertures A through F, respectively. Poisson noise was added to these projections.

The noisy projections were backprojected to create the image vector **B** ^*T*^ **p**. These results were then reconstructed using the MR algorithm. The reconstructed images were compared to the original phantom and the error in each image was calculated. Because this system is large, calculation of the entire singular value spectrum is not feasible. The error was therefore compared only to the condition-number-based noise prediction.

**Figure 4:**
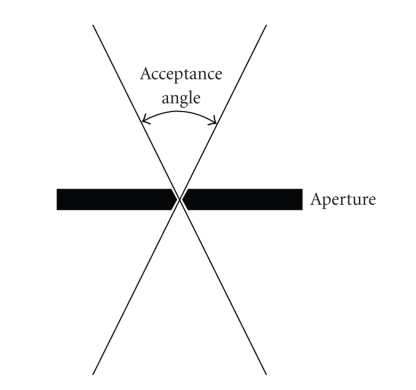
Illustration of acceptance angle.

The acceptance angle of all pinholes in both experiments is 60°, meaning that photons may enter a pinhole at an angle of up to ±30° from perpendicular (Figure 4). In both sets of simulations, the aperture, phantom, and detector were placed as close together as possible while allowing emitted photons from all points in the phantom to pass through the aperture and strike the detector.

### 3.2. Results

**Figure 5:**
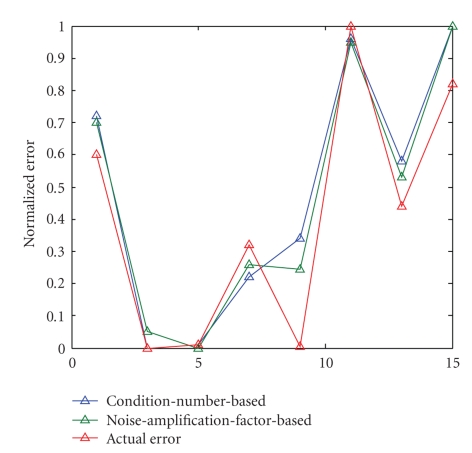
Normalized error predictions and actual error of two-dimensional systems.

The error plots of the preliminary simulations are shown in Figure 5. The plots in Figure 5 show the normalized error predictions based on condition number,

(17) $$\xi_{K}\;=\;\frac{{\left(K(A)\right)}^{2}}{\sqrt{N}},$$

error predictions based on the full singular-value spectrum,

(18) $$\xi_{\text{SVD}}\;=\;\frac{1}{\sqrt{N}}\sqrt{\overset{n}{\underset{k=1}{\sum }}\;\frac{1}{\lambda_{k}^{2}}},$$

and true error, defined as the standard deviation of the error in the image pixels,

(19) $$\xi_{\text{true}}\;=\;\sqrt{\overset{m^{3}}{\underset{i=1}{\sum }}\;{\left(x-A^{-1}B^{T}p\right)}_{j}^{2}}.$$

**Figure 6:**
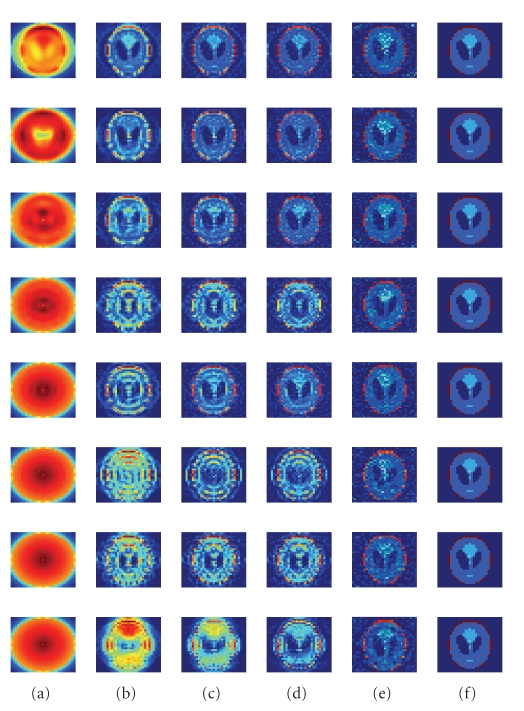
Comparison of reconstructed images in two-dimensional simulation after (a) 1, (b) 9, (c) 17, and (d) 25 iterations. (e) The image reconstructed as **A** ^−1^ **B** ^*T*^ **p**. (f) The original phantom.

The reconstructed two-dimensional images are shown after (a) 1, (b) 9, (c) 17, and (d) 25 iterations in Figure 6. The rows represent results for the apertures with 1, 3, 5, 7, 9, 11, 13, and 15 holes, from the top down. Figure 6(e) shows the image reconstructed as **A** ^−1^ **B** ^*T*^ **p**. The original phantom is shown in Figure 6(f) for comparison. Note that each set of error predictions and of actual errors is normalized; the error predictors, as currently defined, are useful only for comparison between systems and do not represent any absolute real-world value.

**Figure 7:**
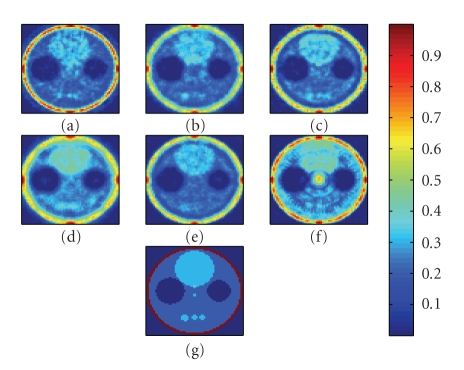
Comparison of reconstructed images from three-dimensional simulations.

**Figure 8:**
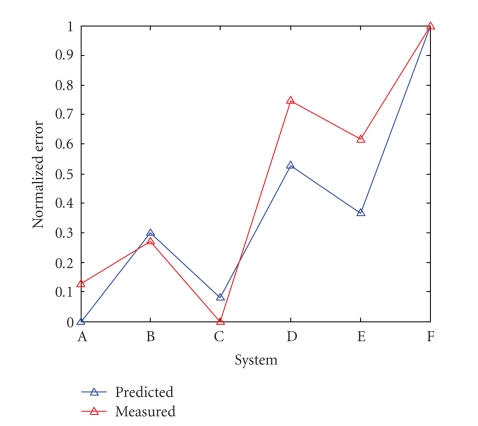
Predicted and measured errors for three-dimensional simulations.

**Figure 9:**
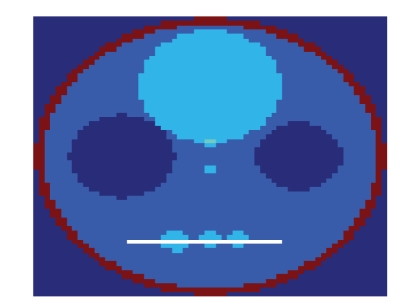
Illustration of line used for profile comparison.

**Figure 10:**
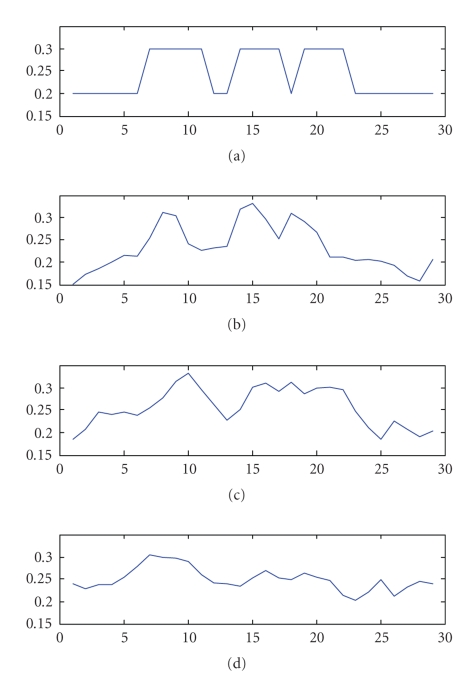
Comparison of system profiles for (a) original phantom, (b) system A, (c) system C, and (d) system E.

The reconstructed images from the final (three-dimensional) simulations are shown in Figure 7. Figures 7(a)–7(f) show slices from the reconstructed images from systems A through F, respectively, at the stage in the MR algorithm at which they are closest to the original phantom (Figure 7(g)). The number of iterations used for the systems is 9 (system A); 11 (system B); 11 (system C); 12 (system D); 20 (system E); 15 (system F). Although most systems use a fixed number of iterations in MR reconstruction, it is not unreasonable to use a different number of iterations for each system. The normalized error predictions for the three-dimensional simulations and the normalized true error are shown in Figure 8. The reconstructed images from the final (three-dimensional) simulations are evaluated both in terms of noise and in terms of lesion detection (an indirect measure of spatial resolution). In order to evaluate the comparative performance of the systems, a composite error is used. This error composite is defined as the sum of the mean squared error of the entire image,

(20) $$\xi_{1}\;=\;\overset{m^{3}}{\underset{i=1}{\sum }}\;{\left(x\;-\;A^{-1}B^{T}p\right)}_{j}^{2}$$

and the square root of the noise power along the profile of the three small lesions in the bottom half of the phantom,

(21) $$\xi_{2}\;=\;\sqrt{\overset{j}{\sum }{\left(x\;-\;A^{-1}B^{T}p\right)}_{j}^{2}}$$

as shown in Figure 9. Sensitivity is measured in the noise parameter, *ξ*_1_, and resolution is measured in the small-lesion profile, *ξ*_2_. Figure 10 compares profiles along the line shown in Figure 9. Using this error composite, the reconstructed images can be evaluated in terms of noise and lesion detection, or sensitivity and resolution.

### 3.3. Analysis

The two-dimensional simulations show the condition number and singular values to be useful in determining relative uncertainty in reconstructed images. Both error estimates ((17) and (18)) perform well in estimating error, but the error estimate which involves the entire singular value spectrum—the noise amplification factor—more accurately predicts system performance. However, the characteristics of any small two-dimensional system are much closer to ideal than those of a real-world system, and are easier to model.

The composite error for the three-dimensional system, as previously defined, was created in order to measure both system sensitivity and spatial resolution. For example, systems A and B are able to resolve the three lesions in the bottom half of the phantom, but contain substantial amounts of noise, as evidenced by the noisy reconstruction of the large bright circle at the top half of the phantom (Figures 7(a) and 7(b)). Systems D and E contain relatively low amounts of noise (Figures 7(d) and 7(e)), but the three small lesions at the bottom are almost indistinguishable. System F (Figure 7(f)) has the worst reconstructed image; although the background is mostly homogeneous, the bright spot at the top is not well defined, and there is a large artifact in the center of the image. This artifact is most likely due to poor placement of pinholes in the aperture. System C is a good compromise between the high-noise problems of the one- and two-pinhole apertures (systems A and B) and the poor resolution of the nine- and ten-pinhole apertures (systems E and F). It also has the lowest error prediction. Note that systems C and D contain the same number of holes, yet have observably different performance, as reflected in the error predictions, the actual composite error, and the reconstructed images.

Note that although the condition-number-based error predictor described in (14) and the composite error measurement described in (20) and (21) show some correlation in this simulation, the error predictor cannot predict exact performance for a particular phantom. The error predictor is derived from the projection and backprojection equations, but has no relation to the phantom in question. It can therefore be used to predict performance generally, but cannot predict performance exactly for a specific phantom. On the other hand, the composite error measurement used above is significant only for this particular phantom, as it relies partially on a profile whose location was specifically selected to match the location of the lesions to be detected. It should be taken as most significant, then, that the systems which performed well in simulation were generally likely to also have low error predictions.

## 4. CONCLUSION

The objective of this research was to create an error estimate that could predict the relative performance of pinhole-based SPECT systems with a reasonable degree of accuracy. To achieve this, the singular value decomposition of the system's projection-backprojection matrix was analyzed. The singular value decomposition allows for a frequency-based analysis, similar to a Fourier analysis. It was based on a function termed the *noise amplification factor*, which is based on the entire singular value spectrum and the photon count of the system. Because of the large amount of computation required to calculate the entire singular value spectrum for a real-world system, a second error predictor was created, based on the condition number and the photon count of the system. However, because the condition number does not contain information from the entire singular value spectrum, it cannot account for the more subtle differences between systems, and is therefore less reliable than the noise amplification factor.

These error predictors were shown to be useful in the prediction of system performance. Six systems with varying numbers and arrangements of pinholes were used to compare predicted and actual errors. The predictions were shown to be useful in determining a preferred system configuration.

The design of a pinhole-based SPECT system is a problem of system design with many variables. The number of pinholes, arrangement of pinholes, detector size and distance from the aperture, acceptance angle, and many other variables all affects the efficacy of an SPECT system in ways that are interrelated. Thus, system optimization cannot be reduced to a combination of single-variable optimizations. Simulation of each possible system configuration is also unfeasible, because of the nearly infinite number of configurations available, and because results will vary depending on the phantom used. For this reason, an unbiased error predictor, based only on the system configuration and not on any empirical data, will provide great benefits to system designers.

A drawback of the SVD-based analysis is the case of an overspecified system. In such a case, the condition number is infinity because the singular values corresponding to high frequencies are zero. In this case, the system resolution must be decreased to a point that all systems under consideration can be analyzed.

The most obvious use for an unbiased error predictor, such as the one described in this paper, is in system optimization. It is therefore the most important of the extensions of this research. However, in order to move to the goal of system optimization, research in this preliminary stage of performance prediction must be expanded.

In the mathematical derivations presented in this paper, Poisson noise was added at the detector. In the frequency-based analysis of the system, this noise was modeled as having equal power at all frequencies. An analysis of the Poisson noise in terms of the singular-value-based frequency spectrum, and incorporation of this knowledge into the error estimate, would add another degree of accuracy to the present error predictions.

When using the MR algorithm for image reconstruction, iteration of the algorithm is terminated after a certain number of iterations. Because of this, high-frequency information is attenuated in the reconstructed image. In order to reflect this in the error predictor, the singular value spectrum must be truncated, as shown in (10)–(12). To do so accurately would require a stronger knowledge of the relationship between the number of iterations performed in the MR algorithm and its effect on the singular value spectrum. It is possible that this relationship can be explained as simply as a high-pass-type transfer function which is applied at each iteration of the algorithm, but it is most likely that the relationship is more complex.

Because calculation of the entire set of singular values for a real-world system is computationally expensive, a function of the condition number was used in this paper to predict system performance. However, it is very unlikely that this is the optimal predictor, even if only using the condition number of the system. The present system could be vastly improved and a detailed system analysis could be made much simpler if a method could be devised to create a rough estimate of the singular value spectrum, or if a better estimate of the noise amplification factor could be derived. If not, a more refined estimation of the noise amplification factor, based on the condition number, would still improve the error estimate somewhat.
